# Prevalent emergence of reciprocity among cross-feeding bacteria

**DOI:** 10.1038/s43705-022-00155-y

**Published:** 2022-08-15

**Authors:** Samir Giri, Ghada Yousif, Shraddha Shitut, Leonardo Oña, Christian Kost

**Affiliations:** 1 Experimental Ecology and Evolution Research Group, Department of Bioorganic Chemistry, Max Planck Institute for Chemical Ecology, 07745 Jena, Germany; 2 Department of Ecology, School of Biology/Chemistry, Osnabrück University, 49076 Osnabrück, Germany; 3 Department of Botany and Microbiology, Faculty of Science, Beni-Suef University, Beni-Suef, Egypt; 4 Present Address: Genome Biology Unit, European Molecular Biology Laboratory, 69117 Heidelberg, Germany; 5 Present Address: Structural and Computational Biology Unit, European Molecular Biology Laboratory, 69117 Heidelberg, Germany

**Keywords:** Microbial ecology, Community ecology

## Abstract

Explaining the de novo evolution of obligate cooperative cross-feeding interactions among bacteria is a fundamental problem. A critical step during this process is the emergence of reciprocity among two interaction partners, because a mutually beneficial exchange of metabolic byproducts can subsequently favour the evolution of cooperative cross-feeding. However, so far, the propensity with which unidirectional cross-feeding interactions transition into bidirectional interactions remains unknown. To address this issue, we systematically cocultured four amino acid auxotrophic genotypes of two bacterial species with potential amino acid donors belonging to 25 different bacterial species. Surprisingly, the results of this experiment revealed that in around 40% of all cases analysed, both the auxotrophic recipient and the metabolically autonomous donor gained a significant growth advantage in coculture. Subsequent experiments clarified that the auxotrophy-causing mutation did not induce the growth-enhancing effect of recipients, but that it was rather due to a generally high propensity of different species to engage in synergistic metabolic interactions. Together, these findings show that reciprocity commonly emerges spontaneously in unidirectional cross-feeding interactions, thus paving the way for the evolution of even tighter metabolic interactions.

## Introduction

Both theoretical and empirical evidence increasingly suggests that bacteria commonly exchange essential metabolites with other members of their microbial community [1–5]. However, it remains usually unclear whether the traded compounds represent byproducts that are inevitably released as a consequence of a cell’s metabolism or if they are produced as cooperative behaviour that has been selected for its beneficial effect on the receiver of the metabolite. This uncertainty is mainly because ancestral populations, from which the identified ecological interaction emerged, are usually not available for experimentation. In addition, it is usually challenging to perform evolution experiments using strains that have been isolated from environmental sources. As a consequence, the prevalence of cooperative cross-feeding interactions in natural microbial communities remains generally unclear and is a matter of intense debate [6].

An important first step in addressing this issue is to identify the conditions that are necessary for cooperative cross-feeding interactions to emerge under controlled laboratory conditions. The knowledge gained in this way can then be used to inform subsequent studies of natural communities.

**Fig. 1:**
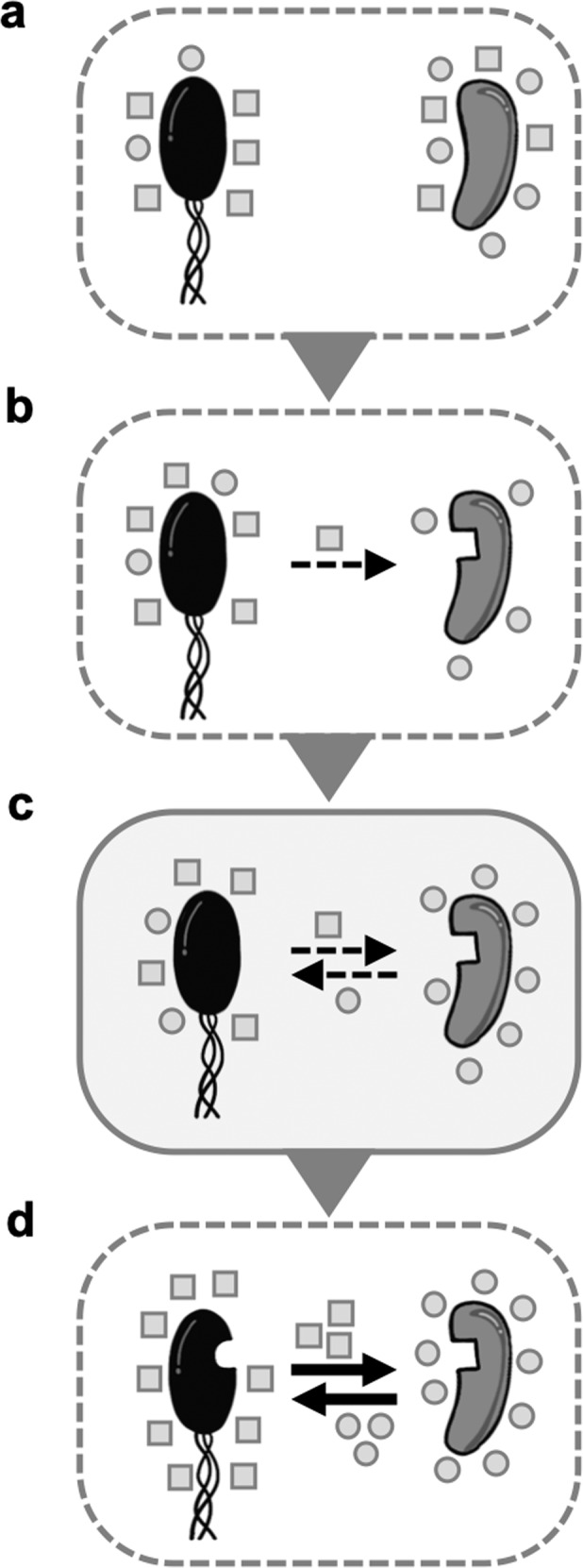
Conceptual model for the emergence of cooperative cross-feeding interactions. **a** Metabolic autonomy: prototrophic donor cells grow autonomously and release metabolites (grey circles and squares) into the extracellular environment. **b** Unidirectional byproduct cross-feeding: auxotrophic mutants arise that have lost the ability to autonomously produce one metabolite that is produced by the other cell. By consuming it, a unidirectional cross-feeding interaction between a prototrophic donor and an auxotrophic recipient is established. **c** Bidirectional byproduct cross-feeding: reciprocity emerges between both interaction partners when the prototrophic donor also starts to benefit from the presence of the auxotrophic recipient. **d** Bidirectional cooperative cross-feeding: a cooperative interaction evolves when both parties start to actively invest costly resources to benefit their corresponding partner. Grey area (**c**) = critical step during the evolution of cooperative cross-feeding, which is the focus of this study. Dashed arrows indicate an exchange of metabolic by-products and solid arrows denote cooperative metabolic interactions. Figure modified after [7].

Based on experimental evidence and theoretical considerations, Pande and Kost proposed a conceptual model to explain the evolution of metabolic cooperation. Their model analyses the evolutionary transition from metabolic autonomy to a situation, in which two bacterial genotypes engage in a reciprocal and cooperative metabolic cross-feeding interaction [7]. For this transition to occur, bacterial cells need to pass through a sequence of at least four different steps (Fig. 1). In the beginning, cells release metabolites such as vitamins or amino acids as unavoidable metabolic byproducts into the extracellular environment (i.e. the *exo-metabolome*) [8–11] (Fig. 1a). Next, certain mutants arise that have lost the ability to autonomously produce some of the released compounds [7] (Fig. 1b). In many cases, these so-called *auxotrophic* genotypes gain a significant growth advantage from consuming the externally available metabolites [2, 5, 12], thus entering into a unidirectional cross-feeding interaction with the provider of these compounds [13]. After that, the initial unidirectional relationship transforms into a reciprocal interaction, for example, by a mutation that closes the interaction loop [2, 7] (Fig. 1c). This last step is critical for the subsequent evolution of mutualistic cooperation (Fig. 1d), in which both parties start to actively invest costly resources to enhance the growth of their corresponding partner [2, 14–16]. This is because positive fitness feedbacks, which result from reciprocal interactions, reward mutants that increase the production of the traded metabolite, even if this investment incurs fitness costs to the producing cell [2, 7, 17–19].

During past years, several experimental and computational studies have corroborated the model of Pande and Kost by providing evidence for the plausibility of many of the proposed intermediate stages of the process. However, the only step, on which very little is known so far, is the emergence of reciprocity from a unidirectional cross-feeding interaction.

Two possibilities are conceivable of how this step can be achieved. First, a mutation in either the donor or the recipient could make the interaction beneficial to the metabolite donor. Alternatively, reciprocity could emerge spontaneously, because donor cells also benefit from some metabolic activity/secretions of their auxotrophic interaction partner. While a mutation causing reciprocity is certainly an important option that should be addressed in the future, here we aim to determine the probability with which reciprocity emerges spontaneously in initially unidirectional cross-feeding interactions between a metabolite donor and an auxotrophic recipient. Assessing the likelihood for this critical step is key for understanding the de novo evolution of mutualistic cooperation, because both theoretical models and experimental evidence suggest that reciprocally beneficial interactions are a fundamental prerequisite for the evolutionary emergence of mutualistic cooperation [11, 20–22]. We address this issue by synthetically assembling pairwise interactions between auxotrophic amino acid recipients and a phylogenetically diverse set of potential amino acid donors. Donor and recipient genotypes belonged to the same or different species and did not share a prior coevolutionary history. Quantifying the growth of both parties under mono- and coculture conditions allowed us to assess the growth consequences resulting for donor and recipient directly and thus determine the chance, with which reciprocity emerges spontaneously in unidirectional cross-feeding interactions.

## Results

Unidirectional cross-feeding interactions were established by coculturing auxotrophic amino acid recipients with prototrophic amino acid donors. To cover a broad taxonomic diversity, 25 different species of four bacterial phyla were selected as potential amino acid donors. To minimise confounding effects that might stem from a shared coevolutionary history, donor strains were selected such that they have not been isolated from the same environment (Table S1). Two auxotrophic genotypes of the two bacterial species (i.e. *Escherichia coli* (EC) and *Acinetobacter baylyi* (AB)) served as amino acid recipients. These mutants were unable to produce one of two amino acids (i.e. histidine (Δ*hisD*) and tryptophan (Δ*trpB*)) and thus essentially required an external source of the respective amino acid to grow.

Using these synthetically assembled interactions, we first determined the number of cases, in which auxotrophic recipients showed detectable growth in coculture with a potential amino acid donor. For this, the growth of auxotrophs in coculture with amino acid donors was compared to its growth under monoculture conditions, in which auxotrophs were cultivated without providing them with the required amino acid. This previously reported analysis revealed that 63% of the 100 cases analysed engaged in a unidirectional cross-feeding interaction in coculture [13]. All instances, in which auxotrophic genotypes did not grow within the focal period of 24 h, were removed from further analysis.

**Fig. 2:**
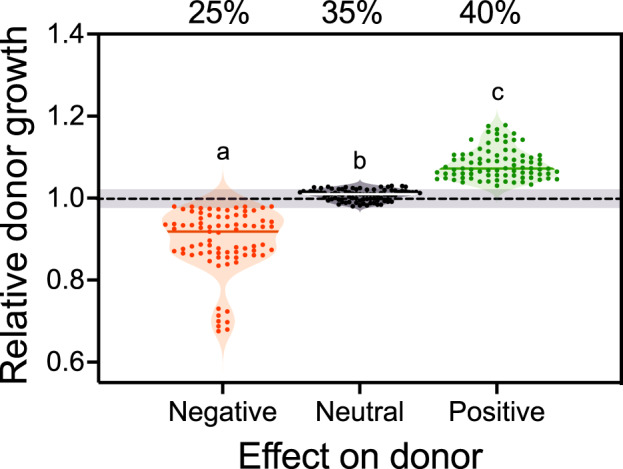
Prototrophic donor cells commonly benefit from the presence of auxotrophic recipients. The growth of donor cells in coculture with auxotrophic recipients is plotted relative to their growth in monocultures. The dashed line corresponds to the growth of donors in monocultures (=1). Values were categorised based on the threshold above (±1.02) and below (±0.98) which donors experience a growth advantage or disadvantage, respectively, in coculture with the recipient (i.e. grey-shaded area). Depending on whether the donor’s growth deceeded or exceeded the threshold, interactions were classified as negative (red, *n* = 60), neutral (black, *n* = 84), or positive (green, *n* = 96). Data points represent replicates and show cumulative results of all comparisons (i.e. two auxotrophies (Δ*hisD* and Δ*trpB)* per two species (*A. baylyi* and *E. coli*)). Violins display the shape and horizontal lines within data points the median of the distribution. Different letters indicate significant differences between groups (Dunn’s post hoc test: *P* < 10^−4^, *n* = 240).

Next, we quantitatively determined how the presence of auxotrophic recipients affected the growth of prototrophic donor genotypes. Analogous to the previous analysis, the growth of donor populations in coculture was compared to their growth under monoculture conditions. This time, however, only those cases were included, in which the recipient benefitted from the presence of the donor (*n* = 240). This analysis revealed that in 25% of experimental combinations, the growth of donors was negatively affected by the presence of auxotrophic genotypes (Fig. 2). This observation is consistent with a cost associated with metabolite cross-feeding. For example, donor cells might release metabolites into the cell-external environment when growing in monoculture, yet consume these nutrients again during later growth stages. However, the presence of auxotrophic cells that continuously consume amino acids likely prevents a re-uptake of released metabolites, which could explain the observed cost of cross-feeding [23]. No significant difference in donor growth between coculture and monoculture conditions was observed in 35% of all cases analysed, suggesting these are neutral interactions that do not affect the donor’s growth. Such neutral effects could indicate that donors did not take advantage of the metabolites the auxotrophs produced, either because of low demand for the corresponding compound or the inability to import them under the given experimental conditions. Alternatively, a growth-enhancing effect of auxotrophs could be counterbalanced by a growth-inhibiting effect, which might be, for example, due to a toxic molecule the auxotroph releases in the presence of the focal donor strain. Surprisingly, 40 % of all donor genotypes tested gained a significant growth advantage when paired with auxotrophic recipients (Kruskal-Wallis test followed by a Dunn’s post hoc test: *P* < 10^−4^, χ^2^ = 221.75, *n* = 240, Fig. 2). Statistically analysing the distribution of negative, neutral, or positive effects revealed a significant effect of the species analysed (two-way ANOVA: *P* < 0.01, F_1, 239_ = 17.04, *n* = 240), but not of the identity of the auxotrophy-causing mutation (two-way ANOVA: *P* = 0.9, F_1, 239_ = 0.005, *n* = 240).

To gain further insights into the rules that determine the spontaneous emergence of reciprocity in the abovementioned experiment, we asked whether the tendency of auxotrophic recipients to enhance or inhibit the growth of cocultured donors depended on the phylogenetic relationship between both partners. No such patterns were detectable in cases, in which donor cells experienced a growth disadvantage when cocultured with an auxotrophic genotype (Spearman rank correlation: *P* > 0.05, *n* ≥ 9, Fig. S1). Interestingly, in those cases, in which donors benefitted from the presence of auxotrophs, we found a significant negative correlation between the phylogenetic distance between donor and recipient and the growth of donor genotypes for both the histidine (Spearman rank correlation: r = −0.49, *P* < 0.019, *n* = 22) and the tryptophan auxotrophic mutant of *E. coli* (Spearman rank correlation: r = −0.754, *P* < 0.0008, *n* = 17) (Fig. S1). However, this pattern could not be detected in the two auxotrophs of *A. baylyi* (Spearman rank correlation: *P* > 0.05, *n* ≥ 21, Fig. S1). Together, this experiment showed that unidirectional cross-feeding interactions commonly result in the spontaneous emergence of reciprocity and that the strength of this effect depends on the species to which the auxotrophic mutant belongs rather than the identity of auxotrophy-causing mutation.

**Fig. 3:**
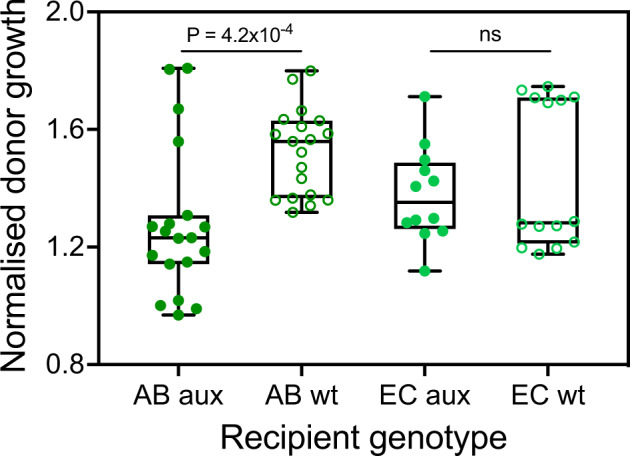
Both auxotrophic and prototrophic genotypes benefit donors in coculture. Cocultures of donor strains with either auxotrophic (aux, filled circles) or prototrophic (wt, empty circles) genotypes of *Acinetobacter baylyi* (AB, dark green) and *Escherichia coli* (EC, light green) reveal significant differences in the growth of donors for *A. baylyi*, but not *E. coli* (Mann-Whitney U-Test: *A. baylyi*: *P* = 4.2 × 10^−4^, Z = −3.401, *n* = 38; *E. coli*: *P* = 0.98, Z = −0.051, *n* = 26). Shown is the normalised donor growth (i.e. growth of donors in coculture minus the donor growth in monoculture divided by the growth of the cocultured auxotrophic or prototrophic recipient after 24 h; see methods). In this experiment, a randomly chosen subset of donor strains was used that previously gained a growth advantage when cocultured with an auxotrophic recipient (Fig. 2). Boxes depict the first and third quartile, the horizontal line the median, and whiskers the 1.5 interquartile range. Data points represent replicates.

The above result motivated us to ask whether the spontaneous emergence of reciprocity was observable in both species that have been used as auxotrophic recipients and, if so, whether the auxotrophic phenotype itself caused this pattern. To answer these questions, prototrophic and auxotrophic genotypes of both species were individually cocultured as recipients with randomly selected donor species that previously benefitted in terms of growth when cocultured with an auxotrophic recipient. Under these conditions, almost all focal donor genotypes gained a significant growth advantage relative to their monoculture controls in all combinations tested (one-sample *t*-tests for AB aux: *P* = 0.01, *t* = 2.6, df = 27; AB wt: *P* = 0.0001, *t* = 5.12, df = 27; EC aux: *P* = 0.049, *t* = 2.04, df = 22). The only exception to this was cocultures that included the prototrophic wild type of *E. coli* as the recipient. In this case, donor growth did not differ significantly from its monoculture controls (one-sample *t*-test: *P* = 0.28, *t* = 1.101, df = 22). However, the growth advantage donors experienced depended on the genotype of the cocultured population in a species-specific manner: in the case of *A. baylyi*, donor growth was significantly enhanced in the presence of prototrophic *A. baylyi* WT cells relative to cocultures with the corresponding auxotrophic genotype (Mann–Whitney U-Test, *P* = 4.2 × 10^−4^, Z = −3.401, *n* = 38, Fig. 3). In contrast, the growth-enhancing effect on cocultured donors did not differ significantly between auxotrophic and prototrophic genotypes of *E. coli* (Mann–Whitney U-Test, *P* = 0.980, Z = −0.051, *n* = 26, Fig. 3). Thus, this experiment corroborated that the prevalent growth advantage amino acid donors experienced in unidirectional cross-feeding interactions was likely not due to regulatory changes, which are induced by the auxotrophy-causing mutation, but rather a property of the cocultured species.

Finally, we asked whether the species-specific growth advantage donors experienced in the coculture experiment was due to metabolites auxotrophic recipients released into the extracellular environment. To address this issue, we collected the supernatant of all four auxotrophic strains as well as of the corresponding wild types. Monocultures of auxotrophic genotypes were cultivated in the presence of the amino acids auxotrophic strains required for growth. Wild type populations were grown in the absence of the amino acids. The resulting cell-free supernatants of auxotrophic or prototrophic genotypes were replenished with fresh media (i.e. 9:1 ratio of supernatant to 5x concentrated MMAB) and supplied to monocultures of donor cells. Subsequently, the growth of donor cells was determined by quantifying population densities after 24 h as optical density at 600 nm. Unsupplemented cultures served as controls. The results of this analysis revealed that metabolites in the culture supernatant could not explain the previously observed growth advantage of amino acid donors. In none of the cases tested did supplementation with the supernatant of a recipient culture enhance the growth of donor genotypes above the levels of unsupplemented controls. While auxotrophic genotypes of both species as well as of the prototrophic wild type of *E. coli* grew significantly less well when supplied with the supernatant of a recipient culture than under unsupplemented conditions (one-sample *t*-tests for AB aux: P = 10^−4^, t = 20.07, df = 46; for EC aux: *P* = 10^−4^, t = 8.228, df = 39; and for EC wt: *P* = 10^−4^, t = 7.137, df = 19, Fig. S2), no difference between both conditions could be detected for cultures of *A. baylyi* WT (one-sample *t*-tests: *P* = 0.376, *t* = 0.9028, df = 23, Fig. S2). These observations suggest that a release of metabolites from auxotrophic recipients alone cannot explain the growth of coexisting metabolite donors. In contrast, other parameters not accounted for in our experimental setup are likely to play a role as well.

## Discussion

In 2017, Pande and Kost proposed a model to explain the evolution of an obligate cooperative cross-feeding interaction starting from two prototrophic bacterial genotypes. Since then, several studies have corroborated the feasibility of three out of four of the suggested main steps required for an obligate cooperative mutualism to evolve. However, empirical support for one of the critical steps was thus far missing: the emergence of reciprocity from a unidirectional cross-feeding interaction. We addressed this issue by analysing synthetically assembled interactions between pairs of different amino acid auxotrophic genotypes and potential amino acid donors. Our results reveal that in an unexpectedly large proportion of cases investigated (i.e. 40%), prototrophic donor cells benefitted from the interaction with auxotrophic recipients (Fig. 2). Strikingly, the observed growth advantage donor genotypes experienced was not causally linked to the auxotrophy-causing mutation, but rather reflected a generally high probability of observing synergistic growth effects in coculture (Fig. 3). Moreover, the growth advantage of donors could not be explained by metabolites auxotrophic recipients released, suggesting another yet unknown mechanism is responsible for enhancing the growth of donors in coculture with the auxotrophic recipients. These findings indicate that reciprocal interactions readily emerge spontaneously, thus paving the way for the evolution of stronger cooperative interactions, in which both parties start to actively invest resources to benefit their corresponding counterpart.

Our experiment revealed that the growth of prototrophic donor genotypes was positively, negatively, or not affected by the presence of auxotrophic recipients (Fig. 2). The observation of an antagonistic effect exerted by a cocultured auxotroph can be explained in several ways. First, when growing in monoculture conditions, donor genotypes may release metabolites in the extracellular environment [8, 24–27] that they utilise again during later stages of growth [23, 28]. However, in coculture with the auxotrophic recipient, these metabolites do not accumulate, but are directly consumed by the cocultured strain [23, 28, 29]. Second, auxotrophic recipients could deplete metabolites in the minimal resource environment, thus arresting the growth of the donor [30]. Third, the auxotrophic strain could release substances (e.g. metabolic waste products) that inhibit the growth of cocultured donors. Indeed, supplementing donor genotypes with the supernatant of recipients resulted in three out of the four cases tested in a significantly reduced growth relative to unsupplemented conditions (Fig. S2), thus providing support for this possibility. All three mechanisms can, either individually or in combination, explain the growth-inhibiting effect of auxotrophs on prototrophic donors and are well in accord with the intuitive notion that competitive interactions, in which organisms enhance their growth at the expense of others, should be common in microbial communities [31].

However, what could explain the unexpected result that prototrophic donor cells can also benefit from the presence of auxotrophic recipients? Our experiment showed that the growth-enhancing effect was not induced by the auxotrophy-causing mutation, but rather a property of the species selected as recipients (Fig. 3). Two possible mechanisms can explain the observed synergistic effects in coculture. The most likely scenario is that the recipient species released a metabolite into the extracellular environment that enhanced the growth of prototrophic donor cells. The released compound could either serve as a nutrient that enhances the growth of the donor when available in a sufficient amount [32] or as a signalling molecule that induces growth-enhancing processes in the donor [1, 24]. The second possibility is more indirect and operates via the distortion of a steady-state by the presence of an auxotrophic or prototrophic recipient. As the donor grows, it releases metabolites into the extracellular environment as an unavoidable consequence of its metabolism [8, 33] or the structure of the bacterial membrane [34, 35]. When growing in monoculture, donor cells will re-import some of the released metabolites, thus reaching a steady-state of production and consumption. If another genotype is present in the same growth environment and starts to consume some of the metabolites that were previously produced by the donor, the latter needs to increase the production rates of the focal compound to maintain its current growth levels. The increased production of the exchanged amino acid and potentially other metabolites could have different side effects (e.g. change in flux through metabolic pathways) [36, 37] that in turn might enhance the growth of donor cells. Our experiments, in which the supernatants of growing recipient cultures were supplied to donor populations, clearly revealed that the amount of metabolite that was present in the late exponential growth phase of recipient populations was not sufficient to explain the growth advantage of donor populations (Fig. S2). However, this does not rule out the possibility that the strong growth advantage observed in donor genotypes was due to a transfer of metabolites from recipients to donors. For example, it is possible that a steady flux of metabolites from recipients to donors could have prevented an accumulation of the exchanged metabolite in the extracellular environment. However, at the moment, the exact mechanism that enhanced the growth of donors in coculture remains unclear. Future work should address this issue and unravel the molecular details underlying this fascinating and important observation.

An interesting detail that emerged in our analysis was the negative statistical relationship between relative donor growth and its phylogenetic relatedness to the cocultured recipient (Fig. S1). Even though this pattern was only observed for one of the two species of auxotrophs tested, it is opposite to previously reported findings, in which the same species of donors and recipients have been analysed [13]. However, both comparisons differed in two important ways. First, the set of pairs analysed in this study was only a subset of the ones that have been included previously [13] (i.e. only combinations, in which the recipient benefitted from the presence of the donor, were included). Second, the growth of donors (this study) and recipients [13] was analysed in a slightly different way to account for the ability of donors but not auxotrophic recipients to grow independently. Nevertheless, this observation suggests that the selection pressures favouring cross-feeding interactions may differ significantly between unidirectional and bidirectional interactions.

In conclusion, the results presented in this study help to explain the evolution of metabolic cooperation between different bacterial species. Our work reveals that the reciprocal exchange of metabolic byproducts between two distinct bacterial genotypes readily emerges spontaneously from initially unidirectional interactions. This finding closes an existing gap in our understanding of how metabolic mutualisms emerge and suggests that explaining their evolution is much less challenging than previously thought [6].

## Methods

### Bacterial strains

Prototrophic bacterial strains belonging to 25 different bacterial species were used as potential amino acid-producing donor populations [13] (Table S1). Two species (i.e. *Escherichia coli* BW25113 and *Acinetobacter baylyi* ADP1) served as recipients that were auxotrophic for either histidine (Δ*hisD*) or tryptophan (Δ*trpB*), resulting in four recipient strains [13]. Genetic targets for deletion leading to auxotrophy of histidine and tryptophan in *E. coli* and *A. baylyi* were identified using the KEGG pathway database [38]. *E. coli* and *A. baylyi* wild type were used as parental strains to construct auxotrophic recipients. In *E. coli*, deletion alleles from existing strains [39] were introduced by P1 transduction [40]. *A. baylyi* mutants were generated using overlap extension PCR [41, 42]. Mutants in *E. coli* and *A. baylyi* were constructed as described [5, 13, 42] (Table S1). In both species, target genes were replaced with a kanamycin cassette to ensure that the corresponding mutants could be isolated from cocultures when plated on agar plates supplemented with 50 µg ml^−1^ kanamycin.

To distinguish different strains in coculture experiments, the β-galactosidase (*lacZ*) gene was introduced into *E. coli* and *A. baylyi* wild type strains. A *lacZ-*containing gentamycin cassette was constructed to label strains with mini-Tn7 elements on plasmid pUC18R6K-mini-Tn7T-Gm-lacZ. To insert gentamycin and *lacZ* in the chromosome, the mini-Tn7 vector was introduced into both prototrophs (*E. coli* and *A. baylyi*) by conjugation involving *E. coli* WM3064, which contained the helper plasmid pTNS2 and *E. coli* WM3064, which contained the donor plasmid pUC18R6K-mini-Tn7T-Gm-lacZ [43]. In brief, *E. coli* and *A. baylyi* strains were grown overnight in 4 ml of lysogeny broth (LB) medium at 30 °C under shaking conditions (200 rpm). The donor and helper plasmid-containing *E. coli* WM3064 strains were inoculated with DAP (diaminopimelic acid) supplemented with ampicillin (100 µg ml^−1^). Next, 0.5 ml of stationary phase culture was washed three times in a microcentrifuge tube with 1 ml LB containing 300 µM DAP to remove the remaining antibiotic. After that, cell pellets of the recipient, helper plasmid-, and donor plasmid-containing strains were resuspended in 250 µl LB containing DAP. A conjugation mixture of all three strains was spotted on a dry LB plate supplemented with DAP and incubated overnight for 18 h at 30 °C. After incubation, cells were scraped off from LB agar plates, suspended in 1 ml LB medium without DAP, and vortexed for 30–60 sec. The whole bacterial culture mix was then concentrated to 100 µl, spotted on LB agar plates containing gentamycin (15 µg ml^−1^), and incubated at 30 °C for colonies to appear. Depending on the recipient strains, transconjugants appeared within 2–3 days of incubation at 30 °C. To further counter-select against *E. coli* WM3064, single colonies were restreaked at least twice on fresh LB plates with gentamycin. Colony PCR was used to verify chromosomal Tn7 insertions in positive colonies using forward (5ʹ ATTAGCTTACGACGCTACACCC) and reverse primers (5ʹ CACAGCATAACTGGACTGATTTC) as described previously [43].

### Culture conditions

All experiments were performed in minimal medium for *Azospirillum brasiliense* (MMAB) without biotin and with glucose (5 g l^−1^) as a sole carbon source [44]. Preliminary experiments showed that all tested species were able to grow in this medium. MMAB medium consists of 3 g L^−1^ K_2_HPO_4_, 1 g L^−1^ NaH_2_PO_4_, 0.15 g L^−1^ KCl, 1 g L^−1^ NH_4_Cl, 5 ml L^−1^ from 60 g L^−1^ solution MgSO_4_·7H_2_O, 0.5 ml L^−1^ from 20 g L^−1^ solution CaCl_2_·2H_2_O, 0.25 ml L^−1^ of 0.631 g 50 ml^−1^ solution FeSO_4_, and trace salts 10 ml L^−1^. 1 L of trace salt solution consisted of 84 mg L^−1^ of ZnSO_4_·7H_2_O, 765 μl L^−1^ from 0.1 M stock of CuCl_2_·2H_2_O, 8.1 μl L^−1^ from 1 M stock of MnCl_2_, 210 μl L^−1^ from 0.2 M stock of CoCl_2_·6H_2_O, 1.6 ml L^−1^ from 0.1 M stock of H_3_BO_3_, 1 ml L^−1^ from 1.5 g 100 ml^−1^ stock of NiCl_2_. The focal amino acids (histidine and tryptophan) were supplemented individually at a concentration of 100 µM. Cultures were incubated at 30 °C under shaking conditions at 220 rpm. Coculture experiments were performed in 96-deep well plates with a culture volume of 1 ml.

### Coculture experiments

All strains were precultured in replicates by picking single colonies from LB agar plates and growing them in MMAB for 20 h. Auxotrophs were precultured in MMAB medium supplemented with the focal amino acids. The next day, precultures were diluted to an optical density of 0.1 at 600 nm as determined using a FilterMax F5 multi-mode microplate reader (Molecular Devices). Approximately 50 µl of these precultures were inoculated into 1 ml MMAB, giving a starting density of 0.005 OD. In cocultures, donor and recipient populations were mixed in a 1:1 ratio by co-inoculating 25 µl of each diluted preculture without amino acid supplementation. Monocultures of donors and recipients without the focal amino acid were used as controls to exclude growth effects in auxotrophs, which could stem from amino acids that have been provided to precultures. All experiments were replicated four times. Cell numbers were determined at 0 h and 24 h as colony forming units (CFU) per ml culture volume by plating the serially-diluted culture on agar plates. Strains were differentiated using selective agar plates. For this, donor strains were plated on MMAB agar plates, whereas auxotrophic recipients were quantified by counting CFUs on LB agar containing the antibiotic kanamycin (50 μg ml^−1^, [13], Tables S2 and S3).

A second coculture experiment was performed to determine whether an auxotrophic phenotype was causal for the growth advantage experienced by donor cells. For this, ≥4 different donor species were randomly chosen for each of the two recipient species from the set used for the first experiment (Table S4). Each selected donor species was inoculated in a 1:1 ratio into pairwise cocultures together with each of the four auxotrophic genotypes or the corresponding prototrophic wild type of *E. coli* and *A. baylyi*. Monocultures of donors as well as of both auxotrophic and prototrophic recipients that were grown without amino acid supplementation served as controls. Each combination was replicated four times and experimental conditions were identical to the previous coculture experiment [13]. The number of CFUs during the onset of the experiment and after 24 h of incubation was determined by plating. To differentiate donors from auxotrophic genotypes, cultures were plated on MMAB agar plates (donor cells) and LB agar with kanamycin (50 μg ml^−1^) (auxotrophic cells). To discriminate the *lacZ*-marked *E. coli* or *A. baylyi* wild type (WT) genotype from donors, populations were plated on MMAB containing 4-bromo-5-chloro-β-indolyl-D-galactopyranoside (X-Gal) and gentamycin (15 μg ml^−1^) (Table S2).

### Phylogenetic distance

The phylogenetic distance between donor and recipient genotypes was calculated as described previously [13]. Briefly, 16 S rRNA gene sequences of all strains were aligned with MUSCLE (EMBL-EBI) and pairwise phylogenetic distances between donor and recipient strains were obtained from a distance-based matrix in MEGA X software. The resulting value quantifies the evolutionary distance that separates the two focal organisms.

### Supernatant experiment

To test whether the growth advantage of donors was mediated by metabolites auxotrophs have released into the extracellular environment, the cell-free supernatants of both auxotrophic (histidine (Δ*hisD*) and tryptophan (Δ*trpB*)) and prototrophic wild type genotypes were harvested and provided to donor strains (Table S4). To extract the cell supernatant, auxotrophs were cultivated with amino acid supplementation (i.e. histidine and tryptophan, 100 μM each) and prototrophic wild types were grown in the absence of amino acid supplementation. All cultures were grown in 2.5 ml MMAB in 48-deep well plates and incubated at 30 °C under shaking condition (220 rpm). In all cases, the strains’ supernatants were isolated in their late-exponential growth phases. The culture was centrifuged for 10 min at 4000 rpm to separate cells from the supernatant. Afterwards, supernatants were filter-sterilised using a 0.22 μm membrane filter (CME, Carl Roth) and the supernatant was replenished with concentrated 5x MMAB with glucose (9:1 ratio of supernatant to 5x MMAB). In the meantime, some of the donors that showed a significant growth advantage in the presence of auxotrophic recipients were grown in 1 ml MMAB in 96-well plates for 18 h (Table S4). After adjusting the donor OD_600nm_ to 0.1, 5 μl of donor culture was added to 195 μl replenished auxotrophic or wild type supernatant in 96-well plates (total volume: 200 μl culture). Four replicates of each treatment and control with and without supernatants were incubated for 24 h at 30 °C under shaking conditions (200 rpm). Growth was quantified by measuring the optical density at 600 nm of cultures at 0 h and 24 h using a FilterMax F5 multi-mode microplate reader (Molecular Devices, Table S3). For each auxotroph supernatant-donor pair or wild type supernatant-donor pair, OD values (i.e. after blank deduction) achieved by donors with supernatants were divided from the values achieved by donor cultures grown without supernatant.

### Normalised donor growth

To determine the effect of amino acid cross-feeding on the growth of donor and recipient strains, the number of CFUs per ml in mono- and cocultures were determined at 0 h and after 24 h. The relative growth of different donors was determined by dividing the growth of each genotype in coculture (CC) by the value it achieved under monoculture (MC) conditions [5, 13]. The normalised donor growth in coculture with either the auxotrophic or prototrophic recipient was then determined as:

Normalised donor growth = ((CFU_Donor CC(24h-0h)_ – CFU_Donor MC(24h-0h)_) / (CFU_auxotrophic or prototrophic recipient CC(24h-0h)_)). The normalised donor growth in coculture was measured as the change in the final donor-to-recipient ratio over 24 h subtracted with the growth donors achieved in monoculture during the same period (Fig. 3).

### Statistical analysis

Non-parametric Kruskal-Wallis tests and Dunn’s post hoc tests were used to identify statistically significant differences between groups (i.e. negative, neutral, and positive effects on donor growth). One-sample *t*-tests were performed to test whether a genotype’s relative growth was significantly different from 1 (i.e. growth of donor monoculture). Non-parametric Mann-Whitney U-tests were applied to identify significant differences in the growth of donors in coculture with either auxotrophic or prototrophic recipients (Table S3). The statistical relationship between relative donor growth and the phylogenetic distance between donor and recipient strains was assessed via Spearman’s rank correlations. All statistical analyses were performed using the SPSS (IBM, version 26) and GraphPad 9 softwares.

## Supplementary information

Supplementary Information

## Data Availability

The datasets generated and analysed during the current study are available from the corresponding author on request.
