# Supplementary material for: Prevalent emergence of reciprocity among cross-feeding bacteria
Source: ISME Commun. 2022 Aug 15;2:71. doi: 10.1038/s43705-022-00155-y (PMC9723789; doi:10.1038/s43705-022-00155-y)
Supplement: Supplementary file 1 — Supplementary Information [file 43705_2022_155_MOESM1_ESM.docx]

**Supplementary information**

**Prevalent emergence of reciprocity among cross-feeding bacteria**

Samir Giri, Ghada Yousif, Shraddha Shitut, Leonardo Oña, and Christian Kost

**Table of contents Page**

Supplemental figures 2

Supplemental tables 4

Supplemental references 9

**Fig S1: Relationship between the relative growth of donors and their phylogenetic distance to the cocultured recipient.** Results are displayed separately for cases in which (a) donor growth was inhibited by the presence of auxotrophs (red, Fig. 2) and (b) donors benefitted from the presence of auxotrophs (green, Fig. 2). Results of Spearman rank correlations for (a): AB his: r = -0.179, P = 0.39, n = 25; AB trp: r = 0.018, P = 0.93, n =29; EC his: r = -0.51, P = 0.16, n = 9; and EC trp: r = -0.14, P = 0.59, n = 17 and for (b): AB his, r = 18, P = 0.43, n = 21; AB trp, r = 0.2, P = 0.33 n =26; EC his, r = -0.49, P = 0.019, n = 22; and EC trp, r = -0.75, P = 0.0008, n = 17. Data points represent replicates and lines are fitted linear regressions.

**Fig S2: Metabolites in the supernatant of auxotrophic and prototrophic recipients cannot explain the species-specific growth advantage of donors.** Donors were supplemented with the supernatant of auxotrophic (aux, fillled circles) or prototrophic (wt, empty circles) genotypes of *A. baylyi* (AB, dark green) or *E. coli* (EC, light green), respectively. Shown is the growth of supplemented donor populations quantified as their optical density at 600 nm relative to the growth levels of unsupplemented populations (= dashed line). The results of one-sample t-test are shown (AB aux: P = 10^-4^ (***), t = 20.07, df = 46; AB wt: P = 0.376 (ns), t = 0.9028, df = 23, EC aux: P = 10^-4^ (***), t = 8.228, df = 39, and EC wt: P = 10^-4^ (***), t = 7.137, n = 19). Boxes depict the first and third quartile, the horizontal line the median, and whiskers the 1.5 interquartile range. Data points represent replicates.

**Table S1: Strains used in this study.**

| Strains | Source | Identifier |
| --- | --- | --- |
| Donors | | |
| *Acinetobacter baylyi* ADP1 (AB) | [1] | [2] |
| *Arthrobacter nicotianae* (AN) | German Collection of Microorganisms and Cell Cultures, DSMZ | DSM 20123 |
| *Agrobacterium tumefaciens* (AT) | Lab stock | N/A |
| *Azospirillum brasilense* (AZB) | German Collection of Microorganisms and Cell Cultures, DSMZ | DSM1690 |
| *Bacillus licheniformis* (BL) | [3] | Soil isolate from sample site coordinates 50.906557, 11.505631 |
| *Bacillus megaterium* (BM) | German Collection of Microorganisms and Cell Cultures, DSMZ | DSM 32 |
| *Peribacillus simplex* (BS) | [3] | Soil isolate from sample site coordinates 50.906557, 11.505631 |
| *Bacillus subtilis* 3610 *comI*^Q12L^ (BSN) | [4] | Provided by Ákos T. Kovács, DTU |
| *Bacillus subtilis* 168 *trpC^+^* (BSS) | [5] | Provided by Ákos T. Kovács, DTU |
| *Cupriavidus metallidurans* (CM) | [3] | Soil isolate from sample site coordinates 50.906557, 11.505631 |
| *Escherichia coli* BW25113 (ECB) | [6] | *E. coli* Genetic resources at Yale CGSC, The *Coli* Genetic Stock Center |
| *Escherichia coli* MG1655 (ECM) | German Collection of Microorganisms and Cell Cultures, DSMZ | DSM 18039 |
| *Flavobacterium johnsoniae* (FJ) | German Collection of Microorganisms and Cell Cultures, DSMZ | DSM 2064 |
| *Nocardia coeliaca* (NC) | [3] | Soil isolate from sample site coordinates 50.906557, 11.505631 |
| *Pseudomonas fluorescens* (PF) | German Collection of Microorganisms and Cell Cultures, DSMZ | DSM 289 |
| *Pseudomonas fluorescense* Pf-5 (PFP) | Lab stock | N/A |
| *Pseudomonas fluorescens* SBW25 (PFS) | Lab stock | [7] |
| *Pedobacter heparinus* (PH) | German Collection of Microorganisms and Cell Cultures, DSMZ | DSM 2366 |
| *Pseudomonas putida* KT2440 (PP) | Lab stock | DSM 6125 |
| *Pseudomonas syringae* pv. tomato DC 3000 (PSD) | Lab stock | N/A |
| *Pseudomonas syringae*subsp. syringae van Hall 1902 (PST) | German Collection of Microorganisms and Cell Cultures, DSMZ | DSM 50315 |
| *Rahnella victoriana* (RV) | German Collection of Microorganisms and Cell Cultures, DSMZ | DSM 27397 |
| *Serratia entomophila* (SE) | German Collection of Microorganisms and Cell Cultures, DSMZ | DSM 12358 |
| *Serratia ficaria* (SF) | Lab stock | Provided by Department of Microbiology, Osnabrück University |
| *Variovorax boronicumulans*(VB) | [3] | Soil isolate from sample site coordinates 50.906557, 11.505631 |
| Recipients | | |
| Auxotrophic genotypes (Aux) | | |
| *Acinetobacter baylyi* ADP1*, ∆hisD::kanR* | [3, 8] | N/A |
| *Acinetobacter baylyi* ADP1*, ∆trpB::kanR* | [3, 8] | N/A |
| *Escherichia coli* BW25113*, ∆hisD::kanR* | [3, 8] | N/A |
| *Escherichia coli* BW25113*, ∆trpB::kanR* | [3, 8] | N/A |
| Prototrophic genotypes (WT) | | |
| *Acinetobacter baylyi* ADP1, lacZ+, Gm^R^ | This study | N/A |
| *Escherichia coli* BW25113, lacZ+, Gm^R^ | This study | N/A |

**Table S2: Reagents and materials used in this study.**

| Reagent or resource | Source | Catalogue number |
| --- | --- | --- |
| Lysogeny broth (LB), Lennox | Carl Roth GmbH | X964.1 |
| Agar-Agar Kobe | Carl Roth GmbH | 5210.2 |
| Dipotassium hydrogen phosphate | Carl Roth GmbH | 26931.263 |
| Sodium dihydrogen phosphate | Carl Roth GmbH | T879.2 |
| Magnesium sulphate heptahydrate | Carl Roth GmbH | P027.2 |
| Potassium chloride | VWR | 26764.260 |
| Calcium chloride dihydrate | Carl Roth GmbH | 5239.2 |
| Ammonium chloride | VWR | 21236.267 |
| Iron (II) sulphate heptahydrate | Merck | 3965 |
| Manganese chloride | AppliChem | A2087.0100 |
| Cobalt chloride hexahydrate | AppliChem | A2087.0100 |
| Boric acid | Carl Roth GmbH | 6943.3 |
| Nickel chloride | AppliChem | A3917.0100 |
| Zinc sulphate heptahydrate | Carl Roth GmbH | K301.1 |
| Copper chloride dihydrate | AppliChem | 131264.1210 |
| Glucose | Carl Roth GmbH | 6887.1 |
| Kanamycin | Carl Roth GmbH | T832.2 |
| Ampicillin | Carl Roth GmbH | K029.2 |
| Gentamycin | Carl Roth GmbH | 0233.2 |
| X-gal (5-bromo-4-chloro-3-indolyl-β-D-galactopyranoside) | Carl Roth GmbH | 3215.4 |
| 2,6-diaminopimelic acid (DAP) | Alfa Aesar | B22391.06 |
| L-Histidine monohydrochloride | AppliChem reagents | A3733,0100 |
| L-Tryptophan | AppliChem reagents | A3445,0100 |
| 48-well deep well plates | Axygen | P-5ML-48-C-S |
| 96-well deep well plates | Eppendorf | 0030506308D |
| Petri dish | Greiner bio-one | 633180 |
| Syringe filters, ROTILABO^®^, 0,22 µm | Carl Roth GmbH | KH54.1 |

**Table S3: Software used in this study.**

| Software | Source | Identifier |
| --- | --- | --- |
| Origin Pro 2017 | OriginLab, Northampton, MA | https://www.originlab.com/index.aspx?go=Products/Origin |
| GraphPad Prism 9 | GraphPad Software, Northside Dr. Suite 560  San Diego, CA | https://www.graphpad.com/scientific-software/prism/ |
| IBM SPSS statistics 26 | IBM Corporation, released 2019. IBM SPSS Statistics, Version 26.0. Armonk, NY | https://www.ibm.com/support/pages/downloading-ibm-spss-statistics-26 |
| Softmax Pro 6 software | Molecular Devices | https://www.moleculardevices.com/en/assets/tutorials-videos/br/getting-started-softmax-pro-6-software#gref |
| MEGA X | [9] | https://www.megasoftware.net/ |

**Table S4: Donor strains used in the auxotroph versus prototroph coculture experiment (Fig. 3 and S2).**

| Species | Genotype | Phenotype | Donor species^1^ |
| --- | --- | --- | --- |
| *Acinetobacter baylyi* ADP1 | Δ*hisD* | Aux | BL, NC, PF, PFP, RV |
| *Acinetobacter baylyi* ADP1 | Δ*trpB* | Aux | BL, NC, PF, SF, RV |
| *Acinetobacter baylyi* ADP1 | WT | Pro | BL, NC, PF, PFP, SF, RV |
| *Escherichia coli* BW25113 | Δ*hisD* | Aux | AZB, PF, PFP, PST, SE |
| *Escherichia coli* BW25113 | Δ*trpB* | Aux | AZB, PF, PST, SE |
| *Escherichia coli* BW25113 | WT | Pro | AZB, PF, PFP, PST, SE |

WT = wild type, Aux = auxotrophic, Pro = prototrophic, ^1^ = for abbreviations see Table S1.

**References**

1. D'Souza G, Waschina S, Pande S, Bohl K, Kaleta C, Kost C. Less is more: Selective advantages can explain the prevalent loss of biosynthetic genes in bacteria. Evolution. 2014;68(9):2559-2570.

2. Vaneechoutte M, Young DM, Ornston LN, De Baere T, Nemec A, Van Der Reijden T, et al. Naturally transformable *Acinetobacter* strain ADP1 belongs to the newly described species *Acinetobacter baylyi*. Applied and Environmental Microbiology. 2006;72(1):932-936.

3. Giri S, Oña L, Waschina S, Shitut S, Yousif G, Kaleta C, et al. Metabolic dissimilarity determines the establishment of cross-feeding interactions in bacteria. Current Biology. 2021; 31, 5547–5557.

4. Konkol MA, Blair KM, Kearns DB. Plasmid-encoded ComI inhibits competence in the ancestral 3610 strain of *Bacillus subtilis*. Journal of Bacteriology. 2013;195(18):4085-4093.

5. Nicolas P, Mäder U, Dervyn E, Rochat T, Leduc A, Pigeonneau N, et al. Condition-dependent transcriptome reveals high-level regulatory architecture in *Bacillus subtilis*. Science. 2012;335(6072):1103-1106.

6. Baba T, Ara T, Hasegawa M, Takai Y, Okumura Y, Baba M, et al. Construction of *Escherichia coli* K-12 in-frame, single-gene knockout mutants: the Keio collection. Molecular Systems Biology. 2006;2(1):2006.0008.

7. Thompson IP, Lilley AK, Ellis RJ, Bramwell PA, Bailey MJ. Survival, colonization and dispersal of genetically modified *Pseudomonas fluorescens* SBW25 in the phytosphere of field grown sugar beet. Nature Biotechnology. 1995;13(12):1493-1497.

8. Oña L, Giri S, Avermann N, Kreienbaum M, Thormann KM, Kost C. Obligate cross-feeding expands the metabolic niche of bacteria. Nature Ecology & Evolution. 2021;5(9):1224-1232.

9. Kumar S., Stecher G., Li M., Knyaz C., and Tamura K. MEGAX: Molecular evolutionary genetics analysis across computing platforms. Molecular Biology and Evolution. 2018; 35, 1547–1549.
